# Anæsthesia by Injection of Chloral into the Veins

**Published:** 1877-04

**Authors:** 


### Anæsthesia by the Injection of Chloral Into the Veins.

According to the *Medical Press and Circular*, MM. Tizzoni and Gracinto Gagliata in the *Revista Clinica di Bologna*, have examined the following points :

1. Is chloral injected into the blood a true anæsthetic ?
2. Is there any serious danger from its use ?
3. What are the risks ?

4. Upon what element does the chloral act?

They have drawn the following conclusions :

1. It is not a true anæsthetic, but a powerful hypnotic. Cutaneous sensibility is not abolished except by large doses. The cornea never properly loses its sensibility.

2. It is dangerous ; it is difficult to measure its action, which varies in different people ; it easily excites phlebitis. It is a poison to the heart.

3. Chloral acts directly on the muscular fibre. It determines contraction of the muscular fibre, and the heart stops in systole.

4. The best means to remedy accidents from chloral is to throw cold water on the head and spine. Pretended antidotes of strychnia, quinine, atropine, and curara are bad.—*Med. and Surg. Rep.*

